# Antifungal activity of schinol and a new biphenyl compound isolated from *Schinus terebinthifolius *against the pathogenic fungus *Paracoccidioides brasiliensis*

**DOI:** 10.1186/1476-0711-9-30

**Published:** 2010-10-12

**Authors:** Susana Johann, Nívea P Sá, Luciana ARS Lima, Patricia S Cisalpino, Betania B Cota, Tânia MA Alves, Ezequias P Siqueira, Carlos L Zani

**Affiliations:** 1 Laboratório de Química de Produtos Naturais, Centro de Pesquisas René Rachou, Belo Horizonte, MG, Brazil; 2 Departamento de Microbiologia, Instituto de Ciências Biológicas, Universidade Federal de Minas Gerais, Belo Horizonte, MG, Brazil; 3 Campus Centro-Oeste Dona Lindu, Universidade Federal de São João Del-Rei, Divinópolis, MG, Brazil

## Abstract

**Background:**

The aim of this study was to isolate and identify the antifungal compounds from the extracts of *Schinus terebinthifolius *(Anacardiaceae) against clinical isolates of the pathogenic fungus *Paracoccidioides brasiliensis*.

**Methods:**

The hexane and dichlomethane fractions from leaves and stems of *S. terebinthifolius *were fractionated using several chromatography techniques to afford four compounds.

**Results:**

The compounds isolated from *S. terebinthifolius *were identified as schinol (**1**), a new biphenyl compound, namely, 4'-ethyl-4-methyl-2,2',6,6'-tetrahydroxy[1,1'-biphenyl]-4,4'-dicarboxylate (**2**), quercetin (**3**), and kaempferol (**4**). Compounds **1 **and **2 **were active against different strains of *P. brasiliensis*, showing a minimal inhibitory concentration value against the isolate Pb B339 of 15.6 μg/ml. The isolate Pb 1578 was more sensitive to compound **1 **with a MIC value of 7.5 μg/ml. Schinol presented synergistic effect only when combined with itraconazole. The compounds isolated from S. *terebinthifolius *were not able to inhibit cell wall synthesis or assembly using the sorbitol assay.

**Conclusion:**

This work reveals for the first time the occurrence of compound **2 **and discloses activity of compounds **1 **and **2 **against several clinical isolates of *P. brasiliensi*s. These results justify further studies to clarify the mechanisms of action of these compounds.

## Background

*Paracoccidioides brasiliensis*, a dimorphic fungus occurring in Central and South America, is responsible for paracoccidioidomycosis (PCM), an endemic disease that could affect at least 10 million people in Latin America [1]. However, notification of its occurrence is not compulsory in the countries where it is endemic, hence accurate figures regarding the disease incidence and prevalence are difficult to determine [2]. PCM is a pulmonary infection characterized by cutaneous and/or mucosal lesions that can disseminate to other organs [3]. This disease constitutes an important health problem in Brazil and had also been found in Venezuela, Colombia, Ecuador and Argentina [1].

Two forms of the disease have been described in the literature: the acute (subacute) juvenile form and the chronic adult form; the former runs a faster course and is more severe than the latter [4]. However, in both cases, cell-mediated immune functions are abnormal and, in the absence of specific therapy, mortality is high. Even if improvements are observed in patients after specific treatment, lesions usually remain as sequels [5]. The disease may develop either directly from a primary focus with no latency period or, more commonly, by reactivation of quiescent infections [1].

The drugs most commonly used for treating patients with PCM are sulfonamides, ketoconazole, itraconazole, and amphotericin B. Sulfonamides were the first class of drugs available for treating patients with PCM, but long periods of treatment may be required (more than 2 years), with increasing concern about drug toxicity, cost of treatment, and unacceptable rates of noncompliance with therapy [1,6,7]. Unfortunately, amphotericin B has been associated with substantial toxicity. Although itraconazole and ketoconazole are effective against *P. brasiliensis*, a long duration of therapy and sometimes a lifelong secondary prophylaxis are required, especially in AIDS patients with high relapse incidence [3].

With the aim of discovering new drug leads that could result in new chemotherapeutic or chemoprophylatic agents to combat *P. brasiliensis *infectitons, our group screened Brazilian medicinal plant's extracts against isolates of *P. brasiliensis *[8]. Among these plants, *Schinus terebinthifolius *showed strong activity against clinical isolates of *P. brasiliensis*. *S. terebinthifolius *Raddi (Anacardiaceae) is a perennial tree occurring in the Brazilian coast. It is known by a variety of common names, e.g., "aroeira-vermelha" and "aroeira pimenteira" [9]. Many biological properties for this medicinal plant were described in the literature: antioxidant and wound-healing [10], antitumoral [9], and antimicrobial activities [11-13,8]. We report herein for first time the investigation of natural products isolated from this plant using in vitro assays with *P. brasiliensis*.

## Methods

### Maintenance of *P. brasiliensis *strains

Five clinical *P. brasiliensis *strains, Pb01 (ATCC- MYA-826), PbB339 (ATCC 32069), Pb18 (Faculty of Medicine, Universidade de São Paulo, São Paulo, Brazil), Pb3 (clinical isolate from chronic PCM, São Paulo, Brazil- MHH Forjaz/TIE Svidzinski), and Pb1578 (clinical isolate PCM, Goiás, Brazil, kindly provided by Dr. Maristela Pereira, Universidade Federal de Goiás) were used in the biological assays. The strains of *P. brasiliensis *were maintained by continuous passages in YPD (yeast, peptone and dextrose) medium at 37°C. The fungi were used after 7-10 days of growth.

### Plant material

The aerial parts of *S. terebinthifolius *(germplasm bank number 44) were collected at EPAGRI (Empresa de Pesquisa Agropecuária e Extensão Rural de Santa Catarina), Itajaí, Santa Catarina, Brazil, in January 2004.

### Extract preparation

The dried leaves (400 g) were extracted by maceration in four liter of ethanol 80% during ten days. After solvent elimination, 36 g of a hydroalcoholic extract (HA-L) was obtained. This extract was then partitioned between water and, successively, with hexane, dichloromethane, and ethyl acetate, resulting in 1.4 g of an hexane fraction (HEX-L), 5.0 g of a dichloromethane fraction (DCM-L), and 6.6 g of the ethyl acetate fraction (EA-L), and 15.2 g of the aqueous fraction (AQ-L). The plant stems (250 g) were submitted to the same procedure resulting in 1.0 g (HEX-S), 3.2 g (DCM-S), 0.8 g (EA-S), and 4.8 g (AQ-S) fractions, respectively [13,8].

### Instruments (General procedures)

Silica gel Merck 60G (70-230 mesh) was used for column chromatography and silica gel pre-coated plates Merck 60F_254 _were used for thin-layer chromatography (TLC). The spots on TLC were visualized under UV at 254 and 366 nm and also after spraying with vanillin-H_2_SO_4 _and heating at 120°C for 10 min. Plates were sprayed with NP-PEG (polyethylene glycol reagent for visualization of flavonoids. Gel filtration was run on columns filled with Sephadex LH-20. Analytical HPLC (high-performance liquid chromatography) were run on a Shimadzu chromatograph equipped with a LC10AD pump, a dual wavelength detector set to 210 nm and 240 nm, and using a Shim-pack C_18 _column (5 μm particle size, 4.6 × 250 mm) at a 1 ml/min flow rate. Semi-preparative HPLC was run on the same equipment described above using a Shim-pack C_18 _(5 μm particle size, 20 × 250 mm) column and 10 ml/min flow rate. Pro-analysis and HPLC grade solvents were purchased from Vetec (São Paulo, Brazil) and Sigma Chemical Co (St. Louis, USA), respectively. The ESI-MS^n ^mass spectra were obtained on an ion trap LCQ Advantage (Thermo Electron, San Jose, CA, USA) mass spectrometer operating in the positive and negative ion modes with an electrospray ionization (ESI) source. The samples were injected directly into the ESI source using the equipment micro syringe. The heated capillary temperature was 200°C, the sheath gas flow rate set to 20 arbitrary units, and spray voltage regulated to 4.5 kV. 1D and 2D NMR (nuclear magnetic resonance) spectra were recorded in CD_3_OD and DMSO-d_6 _on a Bruker Avance DRX 400 MHz spectrometer, using tetrametilsylane as internal standard.

### Chromatographic fractionation of the extracts

HEX-L (1 g) was chromatographed on a column (46 × 3 cm) filled with 60 g of silica gel eluted with mixtures of hexane-methanol of increasing polarity. Seventy-four fractions were collected and tested against *P. brasiliensis *(isolate Pb18). The fractions 10-15 were grouped (26 mg) and fractionated by RP-HPLC on a Shim-pack C_18 _5 μm, 20 × 250 mm column using a gradient of MeOH-water (90-100% of MeOH for 20 min) at flow rate 10 ml min^-1 ^and the absorbance of the effluent measured at 210 and 240 nm. This procedure afforded 15.0 mg of compound **1**.

DCM-S (3 g) was injected in a 46 × 3 cm column containing 200 g of silica gel and eluted with a step-gradient of hexane-methanol of increasing polarity. The collected fractions (127) were combined according to their TLC profile in 37 groups. All groups were tested against *P. brasiliensis *(Pb18) and the majority showed antifungal activity. Group 23 (249 mg) was purified by RP-HPLC in acetonitrile-water gradient from 10 to 50% in 40 min and from 50 to 100% in 10 min, the effluent absorbance was monitored at 220 nm and 240 nm. This experiment yielded three fractions which were subjected to gel filtration in a 30 × 2 cm column filled with Sephadex LH-20 and eluted with MeOH at a flow of 0.5 ml/min. In this way it was possible to isolate 17.0 mg of compound **2 **and 15.0 mg of compound **3 **and compound **4 **(2.6 mg).

### Antifungal activity

#### Determination of minimal inhibitory concentrations (MIC)

The bioassay with all isolates of *P. brasiliensis *was performed following CLSI M27-A_2 _guidelines [14] and modifications suggested by Nakai et al. [15] and Johann et al. [8]. Amphotericin B (Sigma, St Louis, USA) and trimethoprim/sulfamethoxazole (SMT/TMP) (Ducto, Brazil) were included as positive antifungal controls. The concentrations of 25-0.03 μg/ml for amphotericin B and 600-1.17 μg/ml for SMT/TMP were used. Their stock solutions were prepared in DMSO (dimethylsulfoxide) and water, respectively, from which twofold serial dilutions were prepared as described in the CLSI document M 27-A_2 _[14].

#### **Minimal fungicidal concentrations (MFC)**

The *in vitro *minimal fungicidal concentration (MFC) of each compound tested was determined by streaking 10 μl from each well that showed complete inhibition (100% inhibition or a clear well), from the last positive well (growth similar to that of the growth control well), and from the growth control well onto YPD plates. The plates were incubated at 37°C for 7 days only. The lowest drug concentration at which no colonies were able to grow was taken as the MFC value [16].

#### Sorbitol protection assay

MIC values were determined for the *P. brasiliensis *isolate Pb18 using the standard broth microdilution procedure described above. Duplicate plates were prepared: one containing twofold dilutions (from 1000 to 7.8 μg/ml) of compounds **1 **and **2 **and the other containing compounds **1 **and **2 **plus 0.8 M sorbitol. MICs were evaluated after 7 and 10 days of incubation [17].

#### Checkerboard microtiter test

Eight serial twofold dilutions (from 1000 to 7.8 μg/ml) of compounds **1 **and **2 **and amphotericin B (from 1 to 0.007 μg/ml) were prepared with the same solvents used in the MIC test. The checkerboard was prepared in microtiter plate for multiple combinations of two antimicrobial agents. Each row (x axis) in the plate contained the same diluted concentration of the first antimicrobial compound; while the concentration in each subsequent row was half this value. Similarly each column (y axis) in the plate contained the same diluted concentration of the second antimicrobial compound; while the concentration in each subsequent column was half this value. The drug combination in which the growth is completely inhibited was taken as effective MIC for the combination [18].

A 100-μl suspension of Pb18, the same isolate used in the MIC test, was added to each well and cultured for 14 days. Fractional inhibitory concentrations (FICs) were calculated as the MIC of the combination of amphotericin B and compounds **1 **and **2 **divided by the MIC of compounds **1 **and **2 **or AMB amphotericin B alone. The FIC index was calculated by adding both FICs and this was interpreted in the following manner: a synergistic effect when its value is ≤0.5; no interaction when the value falls within the >0.5 to 4.0 range; and antagonistic effect when it is >4.0. This experiment was also performed to determine the effect of a combination of active compounds with SMT/TMP and itraconazole (Janssen-Cilag) [19-21].

## Results

The hexane (HEX-L) and dichloromethane (DCM-S) fractions of crude extracts from stems and leaves of *S. terebinthifolius *were selected for chemical fractionation because they exhibited the strongest activities against isolates Pb01, Pb18, and PbB339 of *P. brasiliensis *and had not presented any toxicity to murine macrophages [8]. The activity of crude extracts of steam and leaves was noted at 30 μg/ml for the three isolates de *P. brasiliensis *aforesaid. For fractions HEX-L the activity was noted at 15.2- 125 μg/ml for the isolates tested and for DCM-S the activity was at 30 μg/ml. However, the fractions EA-S, EA-L, aqueous steam and aqueous leaves did not present good activity with MIC values of 125-1000 μg/ml for EA fractions and 625-1000 μg/ml for aqueous fractions against isolates Pb01, Pb18, and PbB339 of *P. brasiliensis*.

After several chromatographic procedures, compound **1 **was isolated from the HEX-L fraction of *S. terebinthifolius*. Compounds **2**, **3**, and **4 **were isolated from the DCM-S fraction. Their spectral data (ESI-MS, NMR, UV) were obtained; analysis and comparison with data from the literature allowed us to identify **1 **as schinol; **2 **as 4'-ethyl-4-methyl-2,2',6,6'-tetrahydroxy[1,1'-biphenyl]-4,4'-dicarboxylate; **3 **as quercetin; and **4 **as kaempferol (Additional file 1).

**Figure 1:**
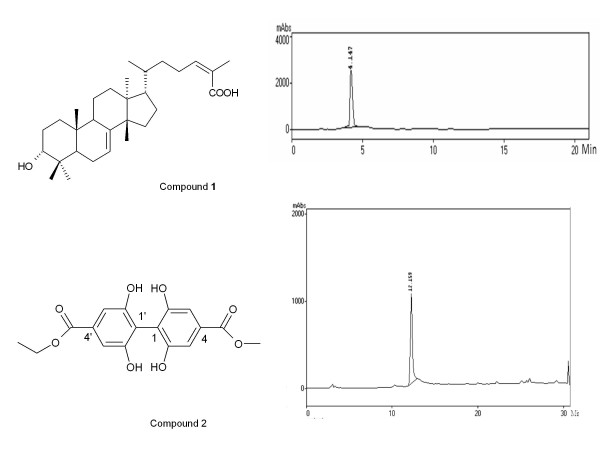
**Antifungal compounds and chromatograms against *Paracoccidiodes brasiliensis *solated from *Schinus terebinthifolius***. Compound **1**: schinol; compound **2**: biphenyl 4'-ethyl-4-methyl-2,2',6,6'-tetrahydroxy[1,1'-biphenyl]-4,4'-dicarboxylate.

Compounds **1 **and **2 **(Figure 1) showed antifungal activity at concentrations ≤250 μg/ml against five isolates of *P. brasiliensis *(Table 1). Compound **1 **displayed activity for the majority of the isolates of *P. brasiliensis*, with MIC values varying from 7.5 to 125 μg/ml. The flavonols **3 **and **4 **were inactive at the highest concentration tested (250 μg/ml).

**Table 1:** Minimal inhibitory concentrations (MIC) compounds isolated from *Schinus terebinthifolius *against different isolates of *Paraccocidioides brasiliensis *(values in μg/ml).

	Pb18	Pb01	Pb3	PbB339	Pb1578
Compound **1**	62.5	125	31.2	15.6	7.5
Compound **2**	250	125	250	15.6	250
Compound **3**	≥250	≥250	≥250	≥250	≥250
Compound **4**	≥250	≥250	≥250	≥250	≥250
Amphotericin B*	0.0062	0.12	0.015	0.0062	0.0062
Trimethoprim-sulfamethoxazol*	300	300	300	75	75

Compound **1**: schinol; compound **2**: 4'-ethyl-4-methyl-2,2',6,6'-tetrahydroxy[1,1'-biphenyl]-4,4'-dicarboxylate; compound **3**: quercetin; and compound **4**: kaempferol. The concentrations of 25-0.03 μg/ml for used for amphotericin B and 600-1.17 μg/ml for SMT/TMP

This is the first time that the antifungal activity of compound **1 **and **2 **is described. The Minimal fungicidal activity was identical to CIM for compounds **1 **and **2**, showing that these compounds have fungicidal activity against *P. brasiliensis*.

Compounds **1 **and **2 **were tested on the isolate Pb18 of *P. brasiliensis *in the presence of sorbitol and showed MIC values similar to those of the medium without sorbitol. Table 2 shows the results of combining known antifungal drugs with compounds **1 **and **2**. When itraconazole was combined with compound **1**, the concentration of the former was halved and that of the latter decreased four times, indicating a synergistic effect (fractional inhibiting concentration index = 0.5) between these substances. No synergy could be observed for the other combinations.

**Table 2:** Synergism activity between compounds 1 and 2 with amphotericin B, trimethoprim-sulfamethoxazole and itraconazole against *Paracocidioides brasiliensis *(isolate Pb18).

Combination	FIC
**Compound 2 **+ amphotericin B	1 (I)^1^
**Compound 2 **+ trimethoprim-sulfamethoxazole	1.5 (I)^1^
**Compound 2 **+ itraconazole	1 (I)^1^
**Compound 1 **+ amphotericin B	1 (I)^1^
**Compound 1 **+ trimethoprim-sulfamethoxazole	1.5 (I)^1^
**Compound 1 **+ itraconazole	0.5 (S)^1^

^1^S, synergy; I, no effect; A, antagonism

Compound **1**: schinol, compound **2**: 4'-ethyl-4-methyl-2,2',6,6'-tetrahydroxy [1,1'-biphenyl]-4,4'-dicarboxylate

## Discussion

In a previous work, our group detected the activity of crude extracts from leaves and stems of *S. terebinthifolius *against clinical isolates of *P. brasiliensis*, *Cryptococcus neoformans*, *Sporothrix schenkii*, and five clinical relevant species of *Candida *[13,8]. The antimicrobial activity of the crude extract from the leaves of *S. terebinthifolius *against *Staphylococcus aureus*, *Bacillus subtilis*, *Pseudomonas aeruginosa*, *Escherichia coli*, and *C. albicans *has been reported elsewhere [12].

According to Johann et al. [13] the leaf extract of *S. terebinthifolius *showed the presence of saponins, flavonoids, triterpenes, steroids, and tannins. Loyd et al. [22] reported the presence of sesqui- and triterpenes. The tetracyclic triterpene schinol (**1**) was first isolated by Kaistha and Kier [23]. It was further isolated from other plant species such as *Juliana adstringens *(Julianiaceae) [24]. We describe here for the first time the isolation and identification of compound **2 **(4'-ethyl-4-methyl-2,6,2',6'-tetrahydroxy[1,1'-biphenyl]-4,4'-dicarboxylate, for NMR data (Additional file 1). This new biphenyl compound is a positional isomer of 4'-ethyl-4-methyl-2,6,3',5'-tetrahydroxy[1,1'-biphenyl]-4,4'-dicarboxylate, a compound already isolated from fruits of *S. terebinthifolius *[25]. This was the first report of the isolation of the flavonols quercetin (**3**) and kaempferol (**4**) from *S. terebinthifolius*. However, both compounds have already been described among species of the genus *Schinus *[26].

When the activity of the compounds isolated in this work is compared to the activity of the extracts and fractions of the *S. terebinthifolius *[8], it is possible to notice that the activity of the **1 **(7.5-62.5 μg/ml) was better than the original fraction HE-L (15.2-125 μg/ml). These results suggest that **1 **could be the main active compound, since the other fractions did not present important activity.

The activity of the original fraction of the compound **2 **was 15.6-125 μg/ml and the crude extract (DCM-S) was 30 μg/ml, thus, other active compounds could be present and by means of synergism, have greater potency than isolated compounds.

The isolates Pb1578 and PbB339 were more sensitive to compounds **1 **and **2**, respectively; they were also more susceptible to SMT/TMP when compared with other isolates of *P. brasiliensis*. Pb01 was the isolate most resistant to compound **1 **(150 μg/ml), while isolates Pb18 and Pb 3 were more resistant to compound **2**. In the present work we tested fungal isolates of *P. brasiliensis *of 2 distinct cryptic phylogenetic species: S1 (Pb18) and PS2 (Pb03) [27]. Pb01 and Pb1578 are known as isolates representative of a new phylogenetic species Pb-01-*like *[28]. Teixeira et al. [28] recommended the formal description of the "Pb-01-like" cluster as the new species *Paracoccidioides lutzii*.

In the present work, compound **4 **(kaempferol) did not present any antifungal activity. However, glucosylated derivates of kaempferol such as 3-*O*-β-D-glycopyranosyl (1→2)-*O*-β-D-glucopyranosil (1→2)-*O*-[α-L-rhamnopyranosyl-(1→6)]-β-D glucopyranoside showed activity against different isolates of *Fusarium oxysporum *[29]. Yordanov et al. [30] showed that kaempferol is a potential inhibitor of the virulent factors (lipolytic and proteinase activities) responsible for the penetration of *C. albicans *into human cells.

Báidez et al. [31] have shown that quercetin (**3**) exhibits activity against *Phytophthora megasperma *and *Cylindrocarpon destructans*. However, in the present work, we found that this compound did not present any activity against *P. brasiliensis*.

Antifungal activity may be due to interference in the cell wall synthesis or assembly. A distinctive feature of compounds acting on the fungal cell wall is that the antifungal effect can be reversed in a medium containing an osmotic stabilizer such as sorbitol. Compounds **1 **and **2 **were tested on the isolate Pb18 of *P. brasiliensis *in the presence of sorbitol and showed MIC values similar to those of the medium without sorbitol. This result suggests that these compounds may be exerting their antifungal activity via mechanisms other than the inhibition of the cell wall synthesis or assembly. Thus, new experiments are needed in order to determine the mechanism of action of these compounds against *P. brasiliensis*.

Use of combined antifungal drugs with different active mechanisms could be a promising therapeutic approach. In our study, we could confirm the antifungal of schinol as well as the synergistic effect between schinol and itraconazole in *P. brasiliensis*. The synergistic effect between schinol and itraconazole might be clinically useful and valuable because itraconazole has a long duration of therapy [3].

## Conclusion

In the present work, the aerial parts of *S. terebinthifolius *furnished two antifungal compounds active on *P. brasiliensis*: schinol (**1**), and new biphenyl identified as 4'-ethyl-4-methyl-2,2',6,6'-tetrahydroxy[1,1'-biphenyl]-4,4'-dicarboxylate (**2**). These natural products will be further studied to evaluate their toxicity and to elucidate their mechanism of action.

## Competing interests

The authors declare that they have no competing interests.

## Authors' contributions

SJ was carried out all experimental work, data acquisition and analysis, literature search and writing the manuscript. NPS was responsible for helpful in assay of antifungal activity. LARSL, BBC, EPS and TMAA involved in characterization of isolate compounds from *S. terebinthifolius*. PSC and CLZ were responsible for study concept, designing and coordinating the research, supervising the work and revising the manuscript. All authors have read and approved the final manuscript.

## Supplementary Material

Additional file 1 **Compounds isolates **Dates of TLC, retention time (*tR*) in HPLC, RMN, UV spectrum and ESI-MS of four compounds isolates. Click here for file
