# Supplementary material for: Antifungal activity of schinol and a new biphenyl compound isolated from Schinus terebinthifolius against the pathogenic fungus Paracoccidioides brasiliensis
Source: Ann Clin Microbiol Antimicrob. 2010 Oct 12;9:30. doi: 10.1186/1476-0711-9-30 (PMC2964558; doi:10.1186/1476-0711-9-30)
Supplement: Additional file 1 — Compounds isolates Dates of TLC, retention time (tR) in HPLC, RMN, UV spectrum and ESI-MS of four compounds isolates. [file 1476-0711-9-30-S1.DOC]

**Additional file 1**

1. **Compounds isolates**

Compound **1**

This substance was obtained (15.0 mg) as a yellow powder. Present spot color brown after spraying with vanillin-H_2_SO_4_,  in TLC, with *Rf* of 0.5 (hexane/AcOet 6:4). Retention time (*tR*) in HPLC: 12.15 min. Its ^13^C NMR DEPT spectra exhibited the presence of twenty five sp^3^ carbons due to seven methyl groups, nine methylenes, five methines including an oxymethine (δ 75.6), four quaternary carbons. Five sp^2^ carbons (δ 142.3; 128.3; 146.5; 118.8) besides carboxyl carbons (δ 170.0). The ^1^H NMR spectrum of **1** showed the presence of five tertiary methyls (δ 0.71, 0.78, 0.82, 0.83, 1.24), a vinyl methyl (δ 1.80) and a secondary methyl [δ 0.86 (d, *J* = 6.0 Hz)] groups, an oxymethine proton [δ 3.23 (brs)] and two vinyl protons [δ 5.22 (brs), δ 5.87 (t, J = 6.0 Hz). Spectra interpretation and comparison with literature data allowed us to identify **1** as schinol.

Compound **2**

This compound was isolated (17.0 mg) as a white powder. Present color brown after spraying with vanillin-H2O4 in TLC, with Rf of 0.62 (MeOH/DCM 8:2). Retention time (*tR*) in HPLC: 4.3 min. Its UV spectrum in MeOH showed peaks at 217 and 275 nm. ESI-MS showed a [M + H]^+^ adduct with *m/z* 348.9 suggesting a molecular formula C_17_H_16_O_8_. In the ^1^H-NMR spectrum, two broad intense signals at δ 6.94 and 6.95 were assigned to H-3,5 and H-3’,5’, respectively. The ethyl group appears as a triplet and a quartet at δ 1.28 and δ 4.21, while the ester methyl group appears as a singlet at δ 3.75. The ^13^C-NMR spectrum of **2** showed eleven signals. The signals at δ 166.31 and δ 165.81 were assigned to two C=O groups. The signals at δ 59.97 and δ 14.25 were assigned to the ethyl group while that at δ 51.56 was assigned to the methyl group. The spectrum also shows two sharp symmetrical signals at 145.55 and 108.5 which were assigned to eight aromatic carbons (C-2,6,2’,6’ and C-3,5,3’,5’), respectively (Table 1). The above assignments were confirmed with HMQC and HMBC experiments. The correlation of the signals of hydrogen at δ 6.94 and 6.95 with the signals of carbons at δ 166.31 and δ 165.81, in the HMBC correlation map, indicate the presence of the OH groups meta to both carboxyl groups. Furthermore, the absence of fragments at *m/z* 318 (M^+^ - 31) and 303 (M^+^ - 45) in the EI-MS spectra is an indication that the OH groups can not be positioned *ortho* to the carboxyl group [1]. From the above analysis, compound **2** was identified as 4'-ethyl-4-methyl-2,2',6,6'-tetrahydroxy[1,1'-biphenyl]-4,4'-dicarboxylate.

Compound **3**

This substance was isolated (15.0 mg) as a yellow solid. Present spot color orange spraying with NP-PEG (polyethylene glycol reagent) in TLC, with *Rf* of 0.32 (DCM/MeOH 9:1). Retention time in HPLC: 15 min. The ESI-MS showed a [M + H]^+^ adduct with *m/z* 302, a value compatible with the molecular formula C_15_ H_10_ O_7_. The ^1^H-NMR spectrum showed five aromatic signals. Two doublets at δ 6.18 and 6.40 (*J* = 1.8 Hz) were due to the *meta* hydrogens in a 5,7-substituted ring A, attributed to H-6 and H-8 respectively. The signals at δ 6.89 (d, *J* = 8.8 Hz, 1H), 7.54 (dd, *J* = 2.0, 8.8 Hz, 1H) and δ 7.67 (d, *J* = 2.0 Hz, 1H) are due to an ABX coupling system and were assigned to H-5′, H-6′ and H-2′. This data is compatible with a flavanol, more specifically, with those published for quercetin [2].

Compound **4**

This natural product was isolated (2.6 mg) as a yellow solid. Present spot color yellow spraying with NP-PEG in TLC, with *Rf* of 0.53 (DCM/MeOH 9:1). Retention time in HPLC: 27.34 min. The ESI-MS showed a [M - H]^-^ adduct with *m/z* 208, compatible with the molecular formula C_15_ H_10_ O_6_. The ^1^H-NMR spectrum showed four signals in the region characteristic of aromatic compounds. Two doublets at δ 6.20 and 6.44 were due to the meta-coupled protons of a 5,7-substituted ring, and were assigned to H-6 and H-8, respectively. The two doublets at δ 6.93 (d, *J* = 8.8 Hz, 2H) and δ 8.05 (d, *J* = 8.8 Hz, 2H) accounted for H-2', H-6' and H-3', H-5' of a 1'-4'-substituted ring. Comparison of the spectral data of compound **4** with those published in the literature showed them to be identical to that of kaempferol [3].

Table 1: ^13^C NMR (100 MHz, DMSO) and ^1^H NMR (400 MHz, DMSO) data for compound **2**.

| Position | δ _C_ | δ _H_; *J* (Hz) |
| --- | --- | --- |
| 1  2  3  4  5  6  1’  2’  3’  4’  5’  6’  CH_2_  CH_3_  CH_3_  -COOMe  -COOEt | 119.59  145.55  108.50  138.38  108.50  145.55  119.30  145.55  108.50  138.30  108.50  145.55  59.97  14.25  51.56  166.31  165.81 | -  -  6.94 s  -  6.94 s  -  -  -  6.95 s  -  6.95 s  -  4.21 q (7.07)  1.28 t (7.07)  3.75s  -  - |

**References**

1. Kim SK, Kim HJ, Choi SE, Park KH, Choi HK, Lee, MW. Anti-oxidative and inhibitory activities on nitric oxide (NO) and prostaglandin E2 (COX-2) production of flavonoids from seeds of *Prunus tomentosa* Thunberg. Arch Pharm Res 2008; 31: 424-428.
2. McLafferty FW. Interpretation of Mass Spectra, 3rd Ed, MillValley, California:University Science Book, 1980.
3. Oh SO, Kim JA, Jeon HS, Park JC, Koh YJ, Hur, H, et al. Antifungal activity of eucalyptus-derived phenolics against postharvest pathogens of kiwifruits. Plant Pathol J 2008; 24: 322-327.
